# The Impact of Abiotic and Biotic Conditions for Degradation Behaviors of Common Biodegradable Products in Stabilized Composts

**DOI:** 10.3390/ma17122948

**Published:** 2024-06-16

**Authors:** Sylwia Stegenta-Dąbrowska, Marek Korendał, Maks Kochanowicz, Marcin Bondos, Paweł Wiercik, Agnieszka Medyńska-Juraszek, Christian Zafiu

**Affiliations:** 1 Department of Applied Bioeconomy, Wrocław University of Environmental and Life Sciences, Chełmońskiego Str 37a, 51-630 Wrocław, Poland; 116146@student.upwr.edu.pl (M.K.); 116145@student.upwr.edu.pl (M.K.); 106652@student.upwr.edu.pl (M.B.); 2 Institute of Environmental Engineering, Wrocław University of Environmental and Life Sciences, Grunwaldzki Square 24, 50-363 Wrocław, Poland; pawel.wiercik@upwr.edu.pl; 3 Institute of Soil Science, Plant Nutrition and Environmental Protection, Wrocław University of Environmentaland Life Sciences, Grunwaldzka Street 53, 50-375 Wrocław, Poland; agnieszka.medynska-juraszek@upwr.edu.pl; 4 Institute of Waste Management and Circularity, Department of Water, Atmosphere and Environment, University of Natural Resources and Life Sciences, Muthgasse 107, 1190 Wien, Austria; christian.zafiu@boku.ac.at

**Keywords:** PLA—polylactic acid, PBAT—polybutylene adipate terephthalate, PBS—polybutylene succinate, FTIR—Fourier-transform infrared spectroscopy

## Abstract

This work examines the influence of the degradation behaviors of biotic and abiotic conditions on three types of biodegradable products: cups from PLA and from cellulose, and plates from sugarcane. The main objective of this study was to evaluate if biodegradable products can be degraded in composts that were stabilized by backyard composting. Furthermore, the impact of crucial abiotic parameters (temperature and pH) for the degradation behaviors process was investigated. The changes in the biopolymers were analyzed by FTIR spectroscopy. This work confirmed that abiotic and biotic conditions are important for an effective disintegration of the investigated biodegradable products. Under abiotic conditions, the degradation behaviors of PLA were observable under both tested temperature (38 and 59 °C) conditions, but only at the higher temperature was complete disintegration observed after 6 weeks of incubation in mature compost. Moreover, our research shows that some biodegradable products made from cellulose also need additional attention, especially with respect to incorporated additives, as composting could be altered and optimal conditions in composting may not be achieved. This study shows that the disintegration of biodegradable products is a comprehensive process and requires detailed evaluation during composting. The results also showed that biodegradable products can also be degraded post composting and that microplastic pollution from biodegradable polymers in soil may be removed by simple physical treatments.

## 1. Introduction

According to the latest market data compiled by European Bioplastics in cooperation with the nova-Institute, global bioplastics production capacities are set to increase from around 2.42 million tons in 2021 to approximately 7.59 million tons per year in 2026 [1]. Compared to the total plastic production of 367 million tons per year in 2020 [2], bioplastics still represent a small fraction of total global production. However, interest in these materials is strongly increasing as indicated by the steadily increasing number of publications on these materials. Biopolymers and bioplastics can be categorized into two major groups, namely biobased and biodegradable plastics. The term “biobased” refers to the origin of the raw materials that were made from biomass and which are at least partially found in the biobased plastic product. However, biobased biopolymers are not necessarily biodegradable, and only a fraction of the known biopolymers undergo the biodegradation process, such as PBAT, PLA, and PBS, while PBAT and PBS are fossil-based products. Jariyasakoolroj et al. (2020) found that biodegradable bioplastics account for 64% of bioplastics [3]. Furthermore, the term “biodegradable” is sometimes misused for marketing reasons as part of the product name of a material, without any actual evidence of its biodegradability [4].

The biodegradation of these plastics depends on the type and dimensions of the product and the prevailing conditions, such as humidity, temperature, pH, salinity, and the presence or absence of oxygen, sunlight, and water. These conditions affect the degradation of abiotic polymers, but also have a decisive impact on the growth and diversity of the microorganism population and the enzymatic activity, especially during the biological decomposition of organic materials, such as during composting [5]. When biodegradable biopolymers are released in the environment, they are initially cleaved into smaller fragments from long polymer chains by non-enzymatic processes, such as photolysis and chemical hydrolysis. These fragments can subsequently be degraded by enzymes produced by microorganisms (e.g., algae, bacteria, or fungi) and can often be ultimately mineralized. The limiting factors of the biodegradation process are the prevailing conditions needed for the growth and reproduction of microorganisms, but the most important factor affecting the rate of biodegradation is the type of polymer, which represents a source of carbon and energy for microorganisms [6].

Favorable conditions for the decomposition of biodegradable polymers are met during the composting process. The decomposition of these polymers through composting can be achieved on the industrial and the domestic scale [7]. However, according to Markowicz and Szymańska-Pulikowska, the largest problem for decomposing biopolymers is that biodegradable bioplastics are often compounded with prooxidative additives [8]. These additives react in an abiotic process to oxidize and cleave the polymer chain. The oxidized fragments can then be biodegraded by microorganisms. However, such additives require distinct conditions (such as sufficient oxygen), which are rarely found in the environment or in home composting, but only in industrial-scale composting facilities. Such situations may lead to the problem that biopolymers are only fragmented under unfavorable conditions and become microplastics, which pose a risk to the health and life of animals and humans due to their heavy metal content and their potential to accumulate in the environment [8]. Therefore, the disposal of bioplastics with organic waste can lead to the same contamination as that from fossil-based plastics. The use of such microplastic-polluted composts can lead to soil contamination [9]. Bioplastics can be a solution to the problem of the increasing amounts of plastics in landfills and the environment, but they must be properly treated, as they can also serve as a nutrition source and can be digested to methane in landfills [10]. In addition, it is necessary to remove bioplastic products from the market, which, despite of the declarations of the producers, decompose only partially or not at all, in order to reduce the risks of microplastic pollution.

This comparative study presents the degradation characteristics of various polymer products typically found in composting facilities. It provides a comprehensive analysis of chemical transformations in controlled laboratory environments, with potential applications in industrial settings.

The purpose of this study was to evaluate the degradation behavior of three commonly available single-use biodegradable products, which are marketed in Poland, labeled “biodegradable” but without certifications and information about specific components, during home and full-scale composting and to identify crucial parameters for degradations behaviors.

## 2. Materials and Methods

### 2.1. Characteristics of Materials

In the experiment, three different marketed single-use products, made from biopolymers, were purchased that are widely and commercially available in Poland. These products were cups made from PLA (polylactide acid) and cups made of sugarcane, as well as plates that were made from cellulose. Detailed information about the composition of the products was not available by the manufacturers. The products were label as “biodegradable”, but not certified. The details of each product are summarized in Table 1. All materials were available online in Poland (Biopack, Poland; https://biopack.com.pl/ (accessed on 15 June 2024)). Each material was cut with scissors into strips of ca. 1 cm width and 7 cm length.

For simulation of the composting process, mature compost from a home composter was used. The composting process was carried out for about two years, and the input to the composter consisted of about 80% food waste and about 20% green waste. The waste was collected from a family of four people living in a detached house with a garden in the city of Wrocław (Poland). For the experiment, the compost was sieved through a <16 mm sieve to homogenize and remove bulky material. The initial moisture of the compost was 62.4% (Table 2).The detailed presentation of the analysis of the compost sample’s physicochemical properties can be found in Section 2.3 and Section 3.1.

### 2.2. Experiment Configuration—Biodegradation Tests

**Figure 1:**
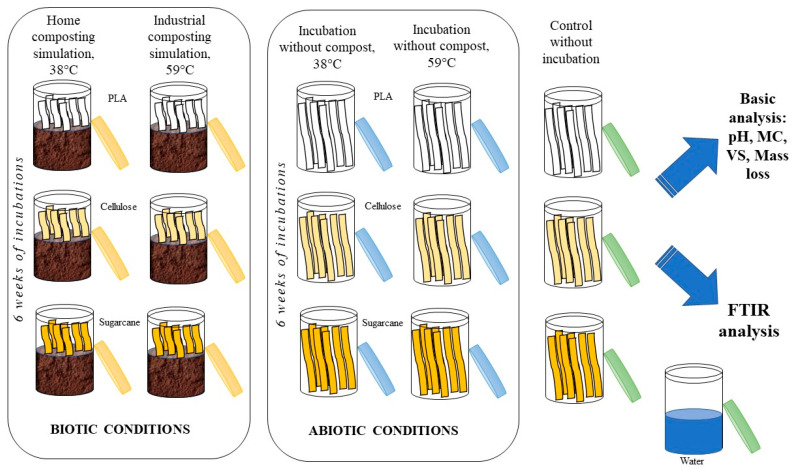
Scheme of degradation behavior tests of biodegradable products. Each vessel contained six strips of polymer material and was prepared in triplicates.

The scheme of the experiment is shown in Figure 1. The compost (ca. 100 g) was placed in 150 mL glass vessels and weighed. The mature compost was utilized to ensure consistent degradation homogeneity. At the same time, empty vessels and biodegradable products were prepared. Six strips of each biopolymer (PLA, cellulose, and sugarcane) were placed vertically in composts (simulation of composting process—biotic conditions) or placed in empty vessels (incubation without compost—abiotic conditions). The strips were lengthened to facilitate material identification for analysis (samples were not completely covered with compost); however, all of the measurements were always performed in the strips covered by compost. All experiments were made in triplicates. Additionally, three containers with compost (blank test) and one vessel filled with 100 mL of water (measurement of the extent of water evaporation) were prepared as controls. Two different temperature conditions were investigated at 38 and 59 °C. For each variant, a set of 16 vessels was prepared and placed in a thermostatic cabinet for 6 weeks. In order to prevent the compost surface from drying out, covers were utilized. These covers were intentionally left slightly ajar to facilitate air circulation. Moreover, the presence of water traps within the thermostatic cabinet served to maintain adequate moisture levels in the surrounding air.

The biodegradable product samples were taken out of each container weekly, gently cleaned with a dry brush, and placed into a string bag, before each vessel was weighed. The collected samples were used for weekly measurements of changes in the chemical structure (FTIR analysis). Compost samples, from the blank sample, were taken at the initial time and after two, four, and six weeks to analyze basic compost parameters: moisture content (MC) and loss on ignition (LOI). At the beginning and end of the experiment, pH and electrolytic conductivity analysis were performed. Each week, gravimetric measurements were made to control the mass loss and evaporation of water from the composting samples. The compost material was also characterized for its content of K, Mg, Na, Ca, P, As, Fe, Co, Mn, Al, Ni, Br, nitrate, and ammonium.

### 2.3. Determination of Physicochemical Properties of Compost Samples

Each compost sample was characterized for its moisture content (MC) after sample drying at 105 °C to a constant mass in a laboratory dryer (WAMED, model KBC-65 W, Warsaw, Poland) according to PN-EN 14346:2011 [11] loss on ignition (LOI) was determined at 550 °C in a muffle furnace (SNOL, model 8.1/1100, Utena, Lithuania) according to PN-EN 15169:2011 [12]. Electrical conductivity (EC) and pH of the compost were measured by CPC-411 meter, (Elmetron, Zabrze, Poland) with a standard procedure by dispersing 10 g of compost in 100 g of deionized water [13]. Reactors were weighed every week to determine water loss by evaporation.

The measurement of the weight loss (to characterize the overall changes during the composting) was performed with a laboratory balance by the following procedure: the empty vessels were weighed first, followed by weighing the vessels with the samples. During the experiment, the measurement was always made after the extraction of biodegradable products strips, which was repeated weekly for a period of 6 weeks for both temperature variants of the experiment. Weight loss was determined by the following:

Weight loss (%) = (W_0_/W_t_) × 100 (1)

W_0_ represents the initial weight (0 weeks) and W_t_ the weight after 1, 2, 3, 4, and 5 weeks.

The cation exchange capacity, which was determined as the sum of base cations, was measured on a microwave plasma–atomic emission spectrometer (MP-AES 4200, Agilent Technologies, Santa Clara, CA, USA) at pH 7.0 after extraction with 1 M ammonium acetate. The heavy metals (As, Fe, Co, Mn, Al, Ni, Br) were measured as a semi-total content after microwave digestion with 10 mL HNO_3_, by the EPA 3051A method also on the MP-AES 4200 [14]. Compost materials were also characterized for their K, Mg, Na, Ca, and P concentrations according to PN-EN ISO 11885:2009 [15]. NO_3_^−^-N andNH_4_^+^-N concentrations were measured according to [16]. The visual identification of biodegradable products at both the commencement and conclusion of the composting process has been successfully demonstrated.

#### FTIR Spectroscopy

Augmented total reflection–Fourier transform infrared (ATR)-FTIR measurements were performed with a Nicolet iN10 integrated infrared microscope with a Nicolet iZ10 external FT-IR module (Thermo Fischer Scientific, Waltham, MA, USA) equipped with a deuterated-triglycine sulfate (DTGS) detector and a diamond ATR module. For each spectrum, 32 scans were averaged in the mid IR range of 400–4000 cm^−1^ at a spectral resolution of 4 cm^−1^. Before FTIR analyses, the biodegradable product samples were cleaned with a brush and dried at 60 °C in a laboratory dryer to exclude humidity that contributes strongly to FTIR spectra. The generated results were compared with the database of the OMNIC^TM^ software suite (ver. 7.0., Thermo Fischer Scientific, Waltham, MA, USA) to exclude external inference. The recognition of tested materials (PLA, cellulose, and sugarcane, respectively) was >98%.

### 2.4. Statictical Analysis

Statistical analysis and graphical interpretation were performed by Statistica 13.3, (StatSoft, Kraków, Poland). Microsoft Excel (ver. 365) was used to plot the FTIR spectra that represent the means of 3 spectra (for each of 32 scans). Spectral subtraction, linear regression (k), and the corresponding coefficient of determination (R^2^) were also calculated in Microsoft Excel.

## 3. Results and Discussion

### 3.1. Compost and Biodegradable Products Characterization

Table 2 shows the properties of the mature compost from the backyard composter that was used as the matrix for the biodegradation tests of biodegradable products in biotic and aerobic conditions. The cation exchange capacity (CEC) was 143.28 cmol(+) kg^−1^, which indicates the formation of carboxylic and phenolic functional groups that are formed by the humification processes and confirms the maturation and stability of the compost [17]. The compost had a slightly alkaline pH of 8.69 and an average conductivity of 3.79 mS cm^−1^, which is similar to the values that were also obtained after the co-composting of food waste and swine manure [18]. The compost exhibited a C/N ratio of 14.7, which is typical for mature composts [16]. Prescott (2010) [19] compared 70 published studies and showed that the C/N ratio (10–12) and total content of litter nutrients had a larger effect on the rate of decomposition. All physicochemical parameters of the compost were in the optimal range for the biodegradation test [20].

The amount of Ca was 28 g kg^−1^ DM, which was less than that in typical mature composts [21]. Lower values were also observed for Na and P, which was attributed to input materials, mainly dominated by food waste and green waste. However, an almost double amount of Mg of 4 mg kg^−1^ DM than typical was also found [22,23]. Additionally, approximately 25% of the total K, Mg, Na, K, and Ca was detected in the leachate, indicating its high suitability for agricultural or horticultural use (Table S1). Furthermore, the concentrations of NH_4_^+^-N and NO_3_^−^-N showed a typical value of about 1000 mg kg^−1^ DM and 100 mg kg^−1^ DM, respectively, which is similar to that of typical composts [24].

The heavy metal content was slightly larger than typical values of green waste, as shown in Table S1 [25]. The alkaline pH and CEC(+) indicated the low mobility of heavy metals, which was also confirmed by the low heavy metal content after leaching (Table S1). Therefore, the compost met the requirements of Polish law for its safe introduction to the soil [25].

The biodegradable products were also analyzed for their MC and LOI (Table 2). The material with the lowest moisture content before the experiment was PLA, with an average content of 1.85%. In contrast, the material with the highest moisture content was sugarcane pulp, with an average moisture content of 4.33%. All materials had more than 95% LOI, which indicated that only low amounts of minerals were present in the samples.

### 3.2. Changes inPhysicochemical Parameters during Composting

**Figure 2:**
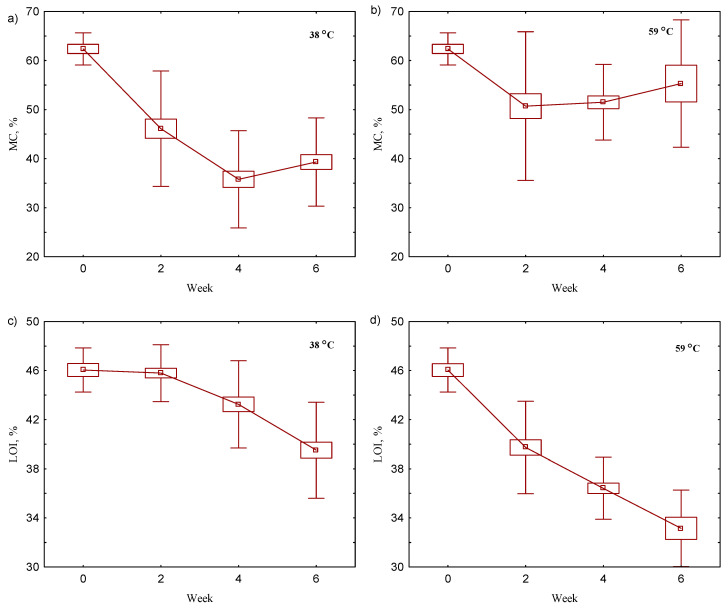
Changes in basic properties of compost during the composting process at 38 °C (**a**) MC/% and (**c**) LOI/%; and at 59 °C (**b**) MC/% and(**d**) LOI/%. The results show the mean values and the error bars indicate the standard error.

During the degradation behavior tests under biotic conditions at 38 and 59 °C, basic physicochemical parameters of the rotting material were measured: bi-weekly MC and LOI (Figure 2) were measured; before and after the total process, MC, LOI, pH, and conductivity were analyzed (Table 3); and weekly mass loss in the reactors was measured (Table 4).

In all cases, the addition of the investigated biodegradable products to the rotting material led to an increased pH (~1.5) after the process compared to the control (Table 3). At the same time, the addition of biodegradable products decreased the EC value (~0.5–1.0 mS/cm). This trend was different from the findings of Adamcová et al., where a slight decrease in pH value was observed during the biodegradation [26]. This finding could have resulted from the different chemical composition of the biodegradable products used in the current study, compared to that of Adamcová et al. Among the compost samples with biodegradable products, the sample of compost with PLA showed the highest pH of 9.28 and conductivity of 5.4 mS cm^−1^ (Table 3), which can be explained by the breakdown of the PLA polymer into lactic acid monomers. The observed properties are typical for the intensive decomposition phase of the composting process, where the pH rises to values of >8 and the conductivity increases to 4–5 mS cm^−1^ [27].

The MC content during the composting process at 38 °C showed a constant loss of water from the samples, from an optimal value of 60% to 45% during 6 weeks of the process (Table 4), which is still a typical value for composts [28]. This was caused by the hydrolysis of organic matter during decomposition, which was confirmed by the loss of LOI during the process (Figure 2) and to a lower extent by the evaporation process (only 1% weight loss, Table 4). Biodegradable products made from cellulose and sugarcane; the moisture content was maintained in the compost at 40% and 48% moisture after the process. In the case of PLA and cellulose, only 40% of the moisture content was found, which was the same value as in the compost without biodegradable products (Table 3). In other research, amounts of 52.8% MC for PLA and 56.0% for starch-based bioplastics (SBB) after composting were recorded [29]. Indeed, the aerobic degradation achieved similar values between the industrial and laboratory scales, despite the moisture levels being significantly different (52.4% and 38.7% for the industrial and laboratory scales, respectively [30]). This result suggests that PLA or its degradation products promoted better conditions for microorganisms involved in the degradation behavior of biopolymers [31]. During the composting, a continuous slow degradation of organic matter was observed (Figure 2), but after the process there were no differences between the different variants of composting with biopolymers—the LOI after 6 weeks was about 40% DM (Table 3).

The initial organic matter content in the compost from the household was found to be 46%. After 6 weeks of composting, the organic matter content decreased to 40% at 38 °C and 35% at 59 °C. This degradation rate is notably lower compared to similar studies, i.e., 63–70% in the semi-pilot scale [32] and 48–50% in the industrial scale after 4 months [33]. At 59 °C, more organic matter (12% DM) was decomposed in the compost than at 39 °C with only about 6% DM after 6 weeks (Figure 2). This indicates that the materials were still not stabilized after the initial backyard composting process. Spaccini et al. also observed a severe decomposition of the original raw material, with a loss of dry mass of 43–50%, under field conditions with dry corn residues and the decomposition of starch-based thermoplastic bio-film [34]. The MC decreased by about 50% and was quite stable towards the end of the process (Figure 2a). This could be a result of high organic matter mineralization to CO_2_ and H_2_O. The mineralization could have directly affected the decomposition of the biodegradable products, which was observed in the samples with biodegradable products, where the MC vs. content was lower at 59 °C than in the experiments at 38 °C (Table S3), which could be explained by more favorable conditions for degradation behavior [35].

Due to the higher temperature, evaporation at 59 °C was higher (35% loss of water) than at 38 °C (2%; Table 4). However, the rotting material that contained biodegradable products made of sugarcane maintained optimal moisture levels, with 45% MC, while PLA and cellulose had moisture levels <40%. The pH was close to 9 and the EC close to 5.7 mS/cm after the rotting process in all samples. Those parameters were larger than those observed in the experiments at 38 °C. This observation was different from that of Janczak et al. (2018), where PLA decreased the pH in soil, which was explained by the ongoing changes in degradation behavior in the PLA, which decomposes to lactic acid that lowers the pH [36]. Therefore, the result of maintaining pH in case of biodegradable products made from PLA indicated that only a minor disintegration was occurring.

### 3.3. Degradation Behavior Composting Tests of Biodegradable Products

Fourier Transform Infrared Spectroscopy (FTIR) was used to study the structural changes in the biodegradable plastic samples. FTIR spectroscopy has been used to describe the composting process and to determine compost maturity, as well as to characterize humic substances in compost or indicate the biodegradation behaviors of biodegradable products [37]. In this study, FTIR analyses were used to determine the chemical changes in the biopolymers PLA, cellulose, and sugarcane by disintegration before and after 6 weeks of rotting in 3 repetitions, as was used before by Wang [38].

#### 3.3.1. Chemical Changes in Biodegradable Products Made from PLA

**Figure 3:**
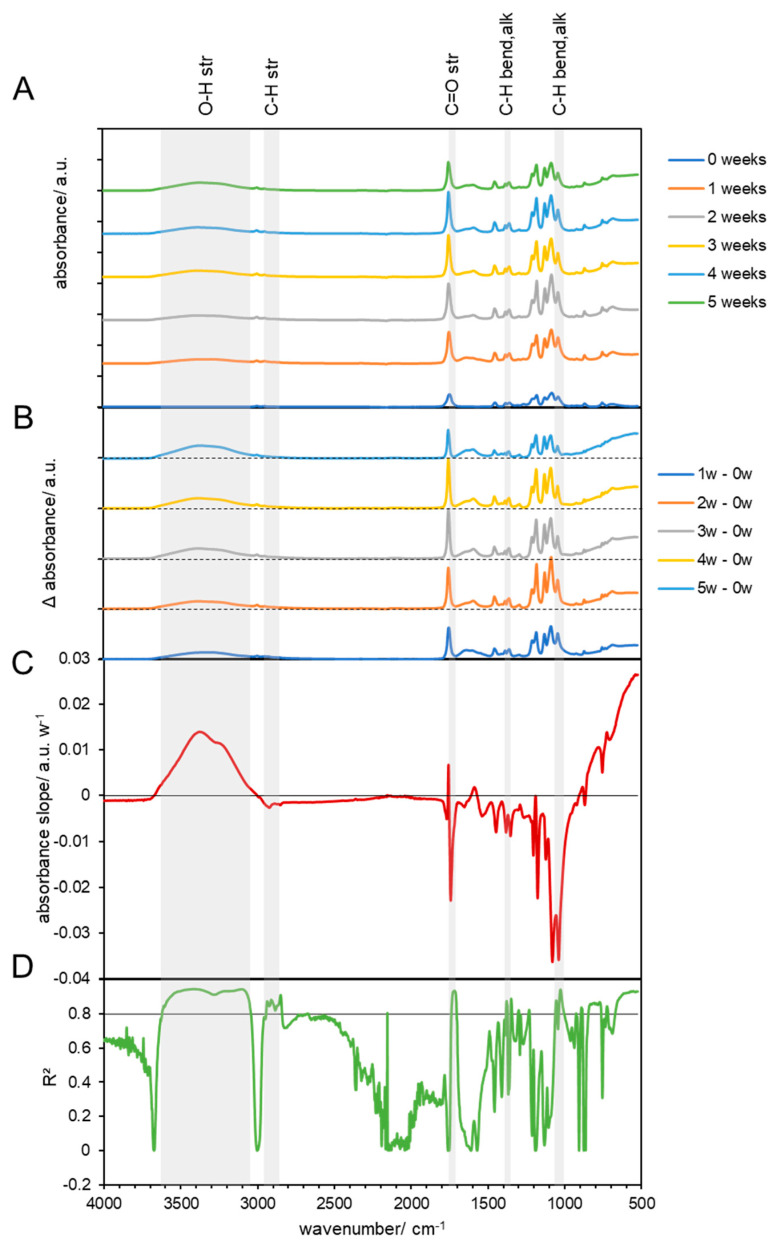
FTIR results of composted PLA at 59 °C. (**A**) FTIR spectra of composted PLA biodegradable products before (0 weeks) and at 1, 2, 3, 4, and 5 weeks after composting. (**B**) Subtraction spectra of samples obtained after 1, 2, 3, 4, and 5 weeks, which were subtracted from the spectra of untreated PLA biodegradable products. The dashed lines indicate zero absorbance change for each subtraction spectrum. (**C**) Linear slopes of the subtraction spectra (**B**) at each wavenumber as the change in the absorbance per week. The horizontal line shows zero absorbance change per week. (**D**) The coefficient of determination (R^2^) of the linear slopes in (**C**). The horizontal line shows a threshold of 0.8. The gray areas that span over all four graphs indicate the wavenumbers of high linearity, with the assignment of the corresponding vibrations on top of the graph, with “str.” for stretching and “alk.” for alkane.

**Figure 4:**
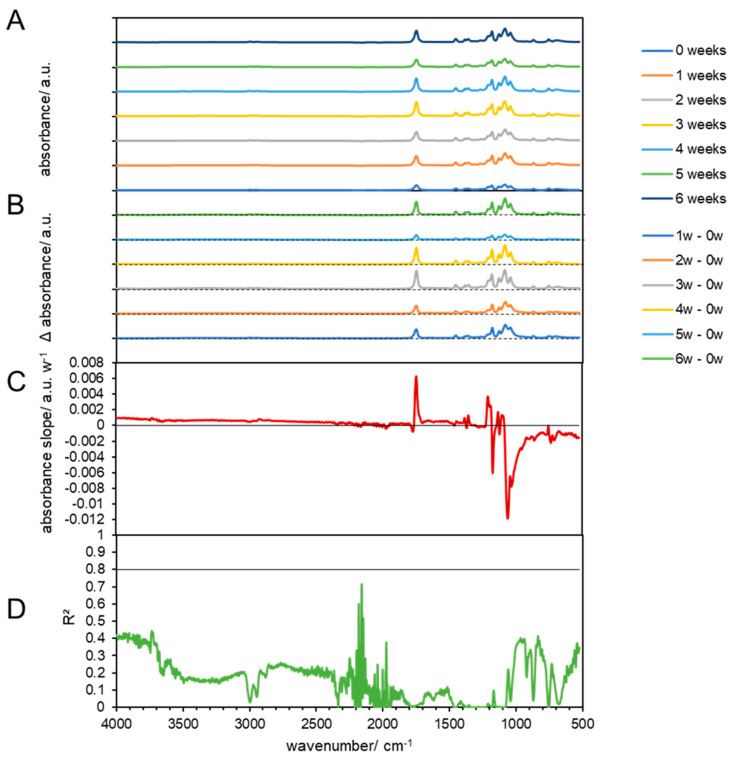
FTIR results of composted PLA at 38 °C. (**A**) FTIR spectra of composted PLA biodegradable products before (0 weeks) and at 1, 2, 3, 4, 5, and 6 weeks after composting. (**B**) Subtraction spectra of samples obtained after 1, 2, 3, 4, 5, and 6 weeks, which were subtracted from the spectra of untreated PLA biodegradable products. The dashed lines indicate zero absorbance change for each subtraction spectrum. (**C**) Linear slopes of the subtraction spectra (**B**) at each wavenumber as the change in the absorbance per week. The horizontal line shows zero absorbance change per week. (**D**) The coefficient of determination (R^2^) of the linear slopes in (**C**). The horizontal line shows a threshold of 0.8.

The FTIR spectrum for pristine PLA biodegradable products showed minor C-H stretching bands at 2997 and 2946 cm^−1^, a large C=O stretching band at 1747 cm^−1^, alkane C-H bending vibrations at 1452 and 1383, and at 1358 cm^−1^, ether C-O stretching vibrations at 1267 and 1209 cm^−1^, C-O-C vibration at 1209, ester C-O stretching at 1181 cm^−1^, C-O stretching (tertiary alcohol) at 1129 cm^−1^, and C-O stretching vibration (secondary alcohol) at 1083 cm^−1^ (Figure 3A and Figure 4A). Below this wavenumber, some other vibrations were observed, which could not be assigned to PLA, but could originate from additives in the material [39]. This considers the peaks at 1745 cm^−1^ and between 3200 cm^−1^ and 3600 cm^−1^ which can correspond to the characteristics of the bond when CaCO_3_ is used as an additive [40]. Additionally, the bands at 2928 cm^−1^ are related to the axial deformation of –CH_2_ bonds in aliphatic chains, associated with the presence of cellulose and lignin [41]. The carbonyl groups C=O (1748 cm^−1^) correspond to the use of glycerol as a plastificator in PLA blends [42]. However, the use of natural additives to PLA, such as Cinnamomum powder [40] or turmeric residue [43], indicated the same bands in the spectrum of the polymer without the additive, indicating that the chemical structure of the matrix did not change with the incorporation of this additive. This is in line with other works, which showed that biodegradable materials consist not only of the pure polymers [44]. In addition, such products need to degrade under variable environmental conditions by the native microorganisms, which may or may not include those that have the ability to break down the polymer [44].

PLA is an aliphatic polyester, and its mechanism of hydrolytic degradation involves the scission of PLA ester groups and the formation of alcohol and carboxylic acid groups [45]. During rotting of the material at 59 °C, more bands appeared, which showed an increasing absorption compared to the pristine PLA and are shown in the subtraction spectra (Figure 3B). The subtraction spectra show that all bands were increasing compared to pristine PLA (week 0) and that a broad band from 3100 to 3700 cm^−1^ appeared, as well as a smaller and also broad band in the range of 1520–1700 cm^−1^. The increase in the broad band at 3100–3700 cm^−1^, which is assigned to -O-H stretching vibrations, indicated that the material was degrading, as more ester bonds of PLA were hydrolyzed. The growth of the bands assigned to hydroxyl bonds with composting time is associated to the main degradation behavior process of PLA under natural conditions by non-enzymatic hydrolysis [46]. The intensity of these bands increased with composting time and was also observed by other authors for PLA biodegradation after 8 days of composting [47]. The broad vibrations at 1520–1700 cm^−1^ cannot be assigned to typical bonds of PLA but may originate from intermediate products of the digestion or oxidation of PLA. In addition, all other characteristic bonds were increasing compared to pristine PLA. However, when the evolution of the changes in the FTIR spectra during the composting process was regarded (vs. 1 week after the composting started), a differentiation between bands with increasing and decreasing absorption was observed. This differentiation and its dependence on the progression of the composting process is presented as the linear slopes of the changing absorption at each wavenumber (Figure 3C). However, the lack of additional data on the absorptivity of the molecules at the different wavenumbers does not allow us to interpret quantitative, but only qualitative, changes, which can be used for a comparative interpretation with respect to other experimental setups in this study. In addition, the coefficient of determination (R^2^) of the linear model was calculated and presented for each wavenumber to highlight the linearity (Figure 3D). A value of R^2^ of >0.8 was selected as the progressive change, which was indicated by the horizontal line in Figure 3D and highlights the characteristic bands that exceeded this threshold by gray areas. The slopes and R^2^ confirm that the -O-H (3100–3700 cm^−1^) increased while -C=O bands (1747 cm^−1^) and -C-H (2997 and 2946 cm^−1^, 1360–1450 cm^−1^, and 1083 cm^−1^) bands were decreasing, which indicates the enzymatic breakdown of PLA due to progressive hydrolyzation of C-H bands and mineralization of the C = O groups. In addition, all other characteristic bands did not change linearly (R^2^ << 0.8), which could indicate that these groups originate from additives that cannot be digested or from intermediates during the biological breakdown process that did not change linearly.

PLA was also incubated for 6 weeks at 59 ° C without compost to investigate the pure temperature in the material. The spectra of the controls showed the same characteristics as those of the pristine PLA, without additional appearing bands (Figure S1A in the Supplementary Materials). Subtraction spectra showed changes in all characteristic bands compared to the pristine material (Figure S1B), which were negative for C=O (1747 cm^−1^) and some C-O bands (1205 cm^−1^), and positive for others at 1172 cm^−1^ and 1066 cm^−1^ (Figure S1B). However, all of these changes had a R^2^ << 0.8, which suggests that the changes were not related to the disintegration of the material but may originate from structural changes (Figure S1D).

When the biodegradable products made from PLA were composted under mild conditions at 38 °C, no new bands appeared in the FTIR spectra within 6 weeks (Figure 3). Changes in the absorbance of bands that were also found in pristine PLA (Figure 4C) showed poor linearity (Figure 4D) and indicated only a possible change in the crystalline and amorphous phases of the material, but no disintegration, as observed at 59 °C. Also, the control experiment without compost showed no linear changes (Figure S2).

Biodegradation behavior changes in PLA were confirmed by FTIR spectrum analysis only at 59 °C and under biotic conditions. The first changes in absorbance became visible after the first week, which could be explained by the initial heating during composting that effected only some PLA strips, which shrank and curled and were also observed before by Solano et al., 2022 [48]. The occurring disintegration was supported by other parameters, such as a good humidity and optimal temperature 59 °C for the bacterial consortium. Other studies showed that PLA disintegration was also possible under mesophilic [49] and thermophilic conditions [50] by specialized organisms. These strains were capable of producing hydrolytic enzymes, such as amylases, cellulases, and lipases. PLA could be degraded only under thermophilic temperature conditions, while mesophilic conditions (38 °C) were not sufficient or these microorganism were not present in the investigated compost. Additionally, in mesophilic temperature conditions, the addition of a special consortium of mesophilic bacteria could be needed for PLA biodegradation [20]. The isolated strain was identified to be *Bordetella petrii* [51]. The addition of specific plant species together with high metabolic activity microorganisms (rhizosphere bacteria and fungi) can accelerate PLA biodegradation [36]. Authors claim that the inoculation of *Miscanthus giganteus* with the *Laccaria laccata* strain could be used for the remediation of soil contaminated with biodegradable plastics, such as PLA. The lack of favorable conditions was also visible by the pH, because PLA is degraded into lactic acid, resulting in a decreasing pH. In this study, we observed an increase in pH at 38 °C and a constant pH at 59 °C during the experiment. This indicates that disintegration at 38 °C, even if it is possible, in this experiment was not supported by microorganisms.

#### 3.3.2. Chemical Changes in Biodegradable Products Made from Cellulose

**Figure 5:**
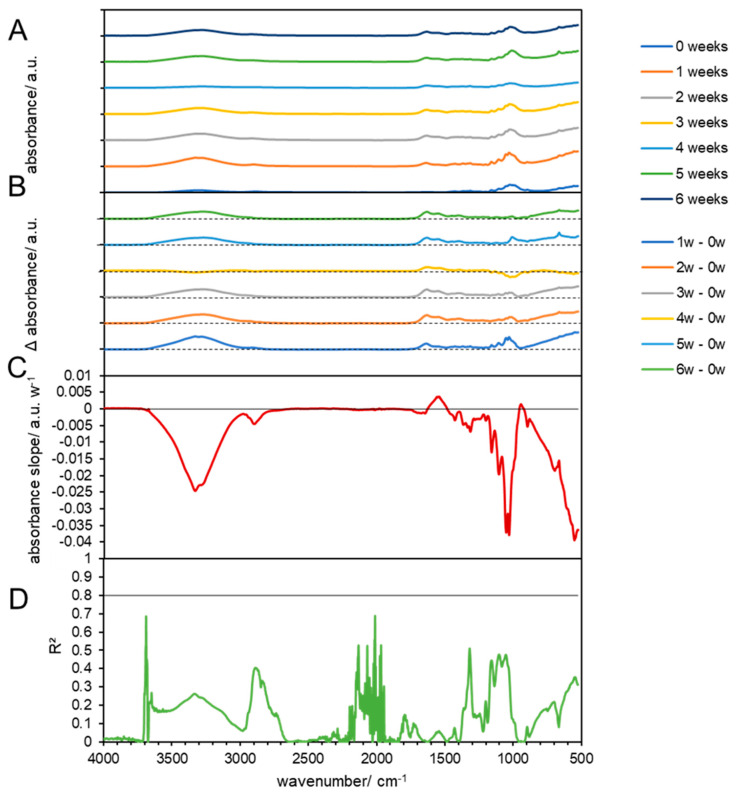
FTIR results of composted cellulose at 59 °C. (**A**) FTIR spectra of composted cellulose biodegradable products before (0 weeks) and at 1, 2, 3, 4, 5, and 6 weeks after composting. (**B**) Subtraction spectra of samples obtained after 1, 2, 3, 4, 5, and 6 weeks which were subtracted from the spectra of untreated cellulose biodegradable products. The dashed lines indicate zero absorbance change for each subtraction spectrum. (**C**) Linear slopes of the subtraction spectra (**B**) at each wavenumber as the change in the absorbance per week. The horizontal line shows zero absorbance change per week. (**D**) The coefficient of determination of the linear slopes in (**C**). The horizontal line shows a threshold of 0.8.

**Figure 6:**
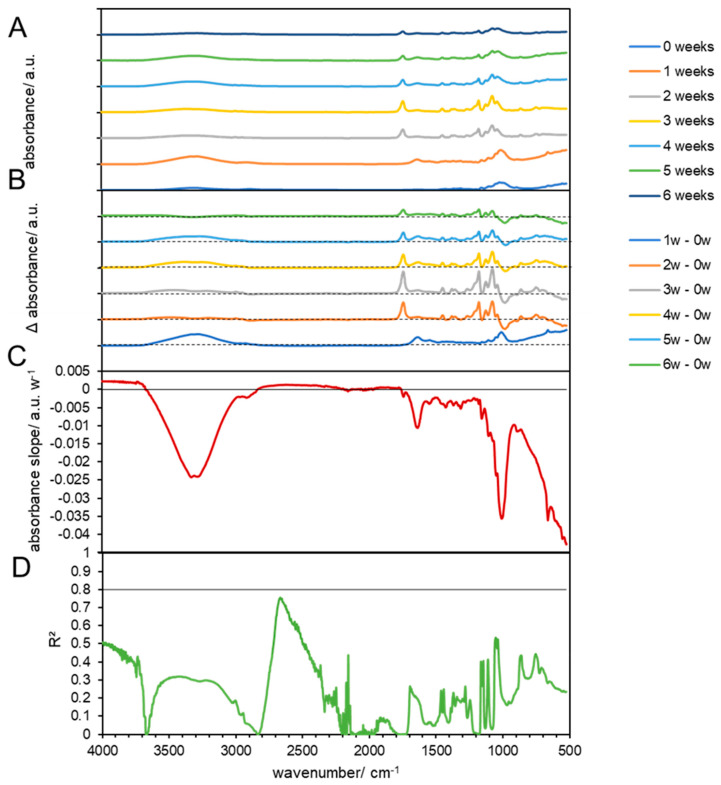
FTIR results of composted cellulose at 38 °C. (**A**) FTIR spectra of composted cellulose biodegradable products before (0 weeks) and at 1, 2, 3, 4, 5, and 6 weeks after composting. (**B**) Subtraction spectra of samples obtained after 1, 2, 3, 4, 5, and 6 weeks which were subtracted from the spectra of untreated cellulose biodegradable products. The dashed lines indicate zero absorbance change for each subtraction spectrum. (**C**) Linear slopes of the subtraction spectra (**B**) at each wavenumber as the change in the absorbance per week. The horizontal line shows zero absorbance change per week. (**D**) The coefficient of determination of the linear slopes in (**C**). The horizontal line shows a threshold of 0.8.

The FTIR spectra of pristine biodegradable products made from cellulose show a broad band from 3100 to 3700 cm^−1^ that is associated with O-H functionalities, as well as a smaller broad band in the range of 1520–1700 cm^−1^, with several maxima, one around 1650 cm^−1^ (surface O-H), and a broad peak with a maximum around 1050 cm^−1^ of -C-O of cellulose (Figure 5 at 58 °C and Figure 6 at 39 °C). Overall, the spectrum matches with the spectrum of microcrystalline cellulose, but exhibits broader bands that can be attributed to the more amorphous structure of the studied material (Figure 5A) [52]. The FTIR spectra of the treated cellulose showed no linear changes in the course of the composting process, which suggests that the material was not degraded via intermediates (Figure 5B–D), which would have been indicated by the appearance of additional bands, but directly mineralized as cellulose is considered to be a fast biodegrading biopolymer [53] and can be degraded by various microorganisms that can be found in composts [54]. The optimal activity lies in the domain of 55 and 80 °C and pH between 5 and 5.5 [55], while composting usually takes places at pH of 8–9 [56]. The 59 °C cellulose incubation in mature compost, a broadening of the hydroxyl group absorbance band (from 3483 to 3280 cm^−1^), was observed (Figure 5). As explained by Gadaleta et al., this broadening indicates the presence of various phenomena: loss of plasticizer, deacetylation and regeneration of the hydroxyl functionalities, hydrogen bonding between the hydroxyl groups of cellulose molecules, and partial degradation of the cellulose backbone [33]. However, the interpretation of the first two phenomena has been complicated by the fact that the spectrum of the plasticizer exhibited peaks overlapping with those of the cellulose [57], which was observed before in PLA in this study. The deterioration of the cellulose structure can also be evaluated by observing the reduction in absorbance peaks at 1035 and 907 cm^−1^. Additionally, there is an increase in peak absorbance at 1621 cm^−1^ due to water absorption from the samples during degradation. Furthermore, a new peak at 1545 cm^−1^ emerges, likely indicating the presence of proteinaceous substances, possibly due to bacterial activity [33].

In addition, no changes were observed when the materials were composted at 38 °C (Figure 5), or only treated by temperature at 59 °C (Figure S3) and 38 °C (Figure S4). These results are in line with earlier studies that showed that heat-treated (at 190 °C) cellulose showed only small changes (<5% in absorbance), particularly in the stretching vibrations (C=O; C-O-C) [58].

#### 3.3.3. Chemical Changes in Biodegradable Products Made from Sugarcane

**Figure 7:**
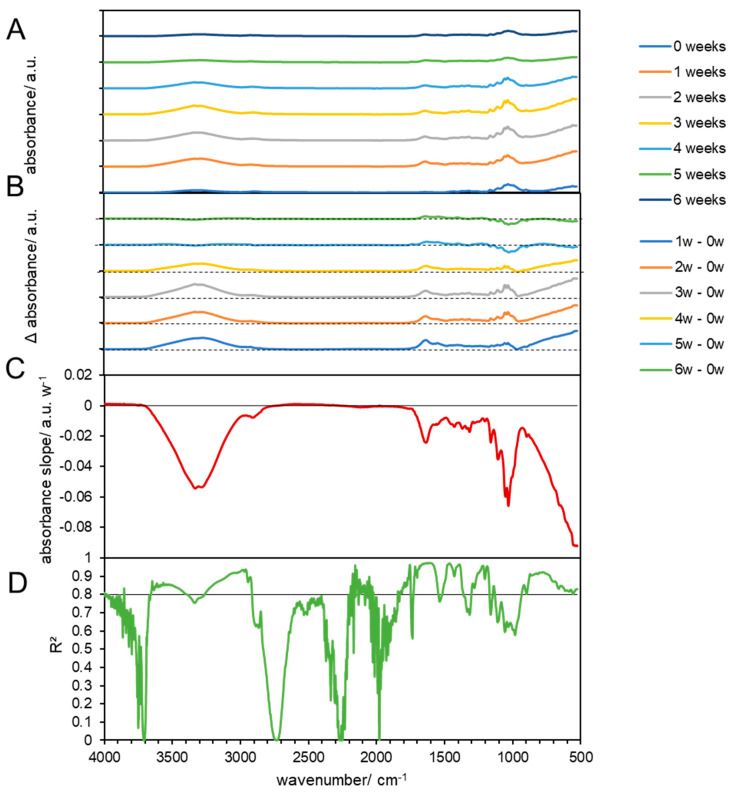
FTIR results of composted sugarcane at 38 °C. (**A**) FTIR spectra of composted biodegradable products made from sugarcane before (0 weeks) and at 1, 2, 3, 4, 5, and 6 weeks after composting. (**B**) Subtraction spectra of samples obtained after 1, 2, 3, 4, 5, and 6 weeks which were subtracted from the spectra of untreated PLA. The dashed lines indicate zero absorbance change for each subtraction spectrum. (**C**) Linear slopes of the subtraction spectra (**B**) at each wavenumber as the change in the absorbance per week. The horizontal line shows zero absorbance change per week. (**D**) The coefficient of determination of the linear slopes in (**C**). The horizontal line shows a threshold of 0.8.

**Figure 8:**
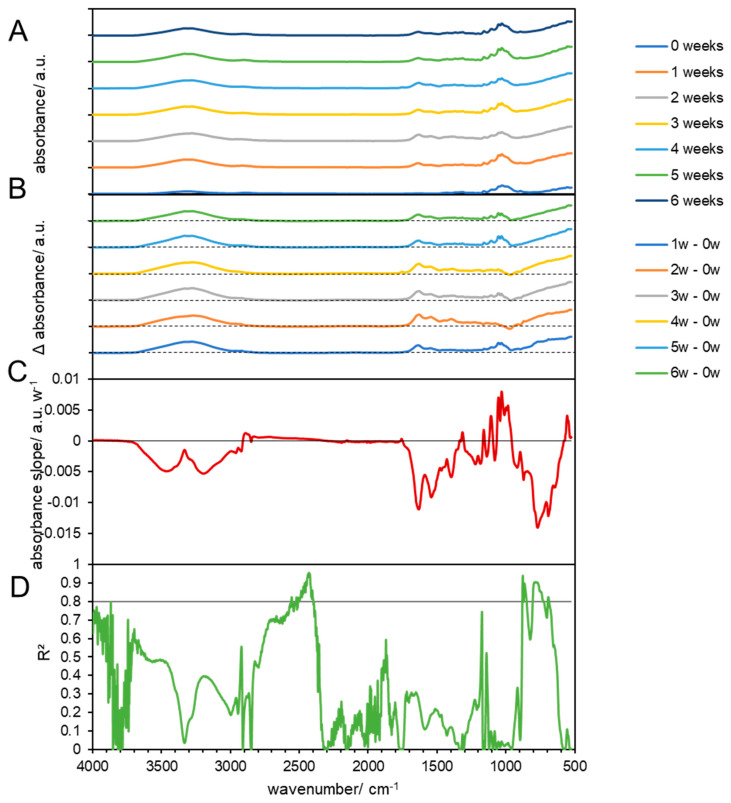
FTIR results of composted sugarcane at 59 °C. (**A**) FTIR spectra of composted biodegradable products made from sugarcane before (0 weeks) and at 1, 2, 3, 4, 5, and 6 weeks after composting. (**B**) Subtraction spectra of samples obtained after 1, 2, 3, 4, 5, and 6 weeks which were subtracted from the spectra of untreated PLA. The dashed lines indicate zero absorbance change for each subtraction spectrum. (**C**) Linear slopes of the subtraction spectra (**B**) at each wavenumber as the change in the absorbance per week. The horizontal line shows zero absorbance change per week. (**D**) The coefficient of determination of the linear slopes in (**C**). The horizontal line shows a threshold of 0.8.

Pristine sugarcane materials are natural materials that are composed of different biopolymers, mainly cellulose, but also lignin and other compounds. The material exhibits spectral bands in the range of 3100–3600 cm^−1^ (O-H stretching), 2800–2970 cm^−1^ (C-H stretching), 1750 cm^−1^ (C-O stretching), 1620–1650, 1510, and 1595 cm^−1^ (the aromatic bands in lignin), 1250 cm^−1^ (C-O in lignin) (Figure 7A) [59].Treatment at 59 °C did not show large differences over time (Figure 7B–D) and therefore it can be expected that the degradation behavior of sugarcane happened more or less in the same way as for cellulose, via direct mineralization and without intermediates. Interestingly, for materials composted at 38 °C, decreases in the absorbance could be observed in sugarcane at all characteristic vibration bands, which indicated that the degradation process was slowed down (Figure 8A–D). Samples that were only heat treated at 59 °C (Figure S5) and 38 °C (Figure S6) showed no changes in characteristic bands.

Similar to cellulose, sugarcane can be considered as an easily biodegradable biopolymer [50]. In this case, sugarcane and cellulose as materials used for the production of cups need to be reinforced with waterproof layers, for the fulfilling the product requirements. These modifications increase the hydrophobicity of sugarcane and decrease the overall degradability of the products. Such additional layers impact the degradation mechanisms of biopolymers by both structural and chemical changes, although the degradation mechanism of sugarcane should be similar to that of cellulose [60]. However, the biodegradation of cellulose products also depends on the material and its structure. For example, fibers or powders have faster degradation rates compared to larger and more dense materials [56]. The extended durability of these materials suggests that their substantial thickness (>1 mm) hinders effective disintegration, as previously observed in the biodegradation of coffee capsules [33]. This highlights the importance of considering geometric factors in biodegradation research, which are often not fully anticipated in small-scale disintegration and biodegradation experiments.

### 3.4. Effect of Abiotic and Biotic Parameters on Disintegrationof Biodegradable Products Made from PLA, Cellulose, and Sugarcane

**Figure 9:**
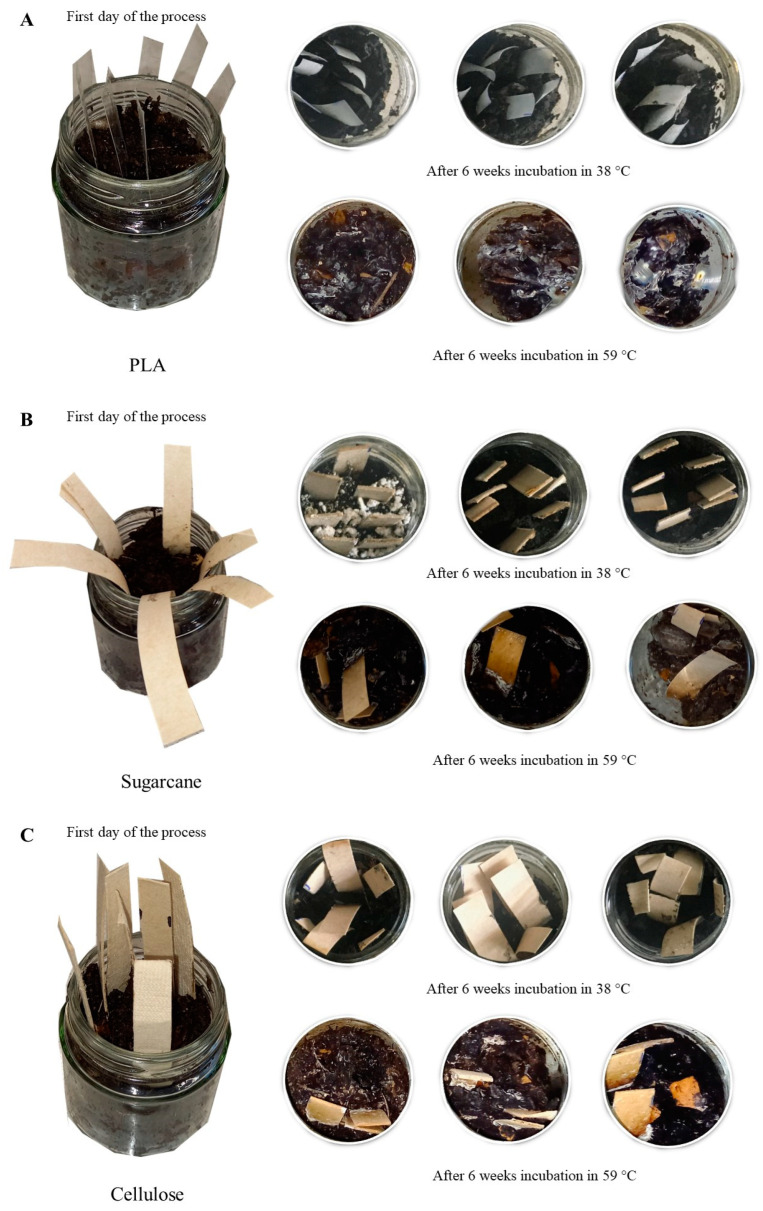
Visual identification of biodegradable products in 38 and 59 °C s: (**A**)—PLA, (**B**)—sugarcane, (**C**)—cellulose.

Biotic factors were demonstrated to have the dominant influence on the biodegradability of bio-based polymers [37]. The main abiotic factors that accelerate the biodegradation in composting are temperature and pH. Our study confirms that, under the conditions that were studied, abiotic conditions act only in combination with biotic factors—incubation of the biodegradable products in mature composting. The effect of temperature was not enough to induce degradation of the chemical structure of biodegradable products. A comparative analysis of the most common biopolymers by Cucina et al. (2021) [61] showed that these polymers were rapidly biodegraded under industrial composting conditions and that the degradation rate continuously increased in the following order: PLA blends < starch-based blends ≤ PHA blends. Consequently, the estimated time of complete degradation of bioplastics was 84 ± 47 days, 124 ± 83 days, and 119 ± 43 days for PLA, PHA, and starch-based blends, respectively [61]. However, composting is maintained in most cases for 90 days, which is less than the degradation time for most bioplastics, which was >90 days [61]. Visual inspection (Figure 9) proved that only PLA in 59 °C sufficiently achieved full biodegradation—after 6 weeks, only scraps on the surface were still noticeable. The sugarcane and cellulose were still fully visible in the compost matrix.

In order to test the biodegradation of biopolymers in the composting process, various mixtures of organic waste were used, with optimal input parameters, i.e., C/N ratio in the range of 1:20 to 1:30 and humidity of about 45–60% (Table 5). Particular attention should be paid to the pH of the input materials, which should be slightly alkaline (~7.5–8.5), because during the decomposition of biodegradable films (the presence of soluble acids), the pH may decrease and the composting process may be inhibited [62]. This is less important when mature compost is used as substrate, because of its cation exchange capacity CEC(+), which compensates for pH changes. However, most of the researchers used mixtures of different wastes to test the biodegradation (Table 5). Therefore, these parameters should be considered as a key parameter, especially in PLA biodegradation. A low pH in the input material could be a simple indicator for microorganisms that are capable of biodegrading cellulose effectively, but this aspect needs to be more evaluated in the future. In this study, PLA biodegradation also showed the easiest biodegradation, which could be explained by the most favorable conditions for these biodegradable products. We suppose that cellulose biodegradable products need more acidic conditions to support thermophilic fungi [56].

Any claim of compostability or biodegradability should be clearly related to the conditions under which biodegradability is investigated. This study confirmed the statement of previous researchers that even when the producent confirms the biodegradability, the same conditions are not easy to repeat under realistic conditions. The FTIR analysis also confirmed that additional chemical components were added to biodegradable products to support their functional properties. Those unknown substances were used to increase the resistance and hydrophilicity of products made from cellulose and sugarcane, which also represent a barrier for microorganisms and their enzymes, which was also shown in this study. In addition, PLA materials did not show spectra of the pristine material, which also confirmed that the product mass was made from blends, which were not specified by the manufacturer. However, in general, the PLA had better water resistance properties, probably because it had less additives, which helps with better biodegradation.

In this research, we observed that the biodegradation of broadly marketed biodegradable products in Poland is challenging to be treated by composting. For this reason, we agree with Solano et al. that these bioplastics should not be marketed until they are confirmed and certified for biodegradability or compostability by common standard test schemes (e.g., EN13432) [48].

## 4. Conclusions

This study shows that the disintegration of products made from biopolymers and marketed as “compostable” needs a detailed evaluation under realistic conditions. The chemical compositions and thickness of products made from biopolymers and the use of functional additives are very broad and can strongly affect their biodegradation behavior. These factors should be measured each time when new blends are introduced to the market (e.g., by testing their biodegradability). The effect of chemical additives to increase the water resistance in cellulose and sugarcane was strongly observable. In these cases, changes in the form of composting application for fiber or powder could help in increasing the biodegradation rate. These results would need additional processes like grinding to be required before the composting. Different conditions of composting, such as an acidic pH, could also help to support mesophilic fungi, which excrete more potent enzymes and are capable of degrading such polymers faster.

Abiotic and biotic condition cooperation is needed for an effective degradation of the investigated biodegradable products. Heat alone is not sufficient to promote the disintegration of PLA, cellulose, and sugarcane. Under abiotic conditions, the biodegradation behavior of PLA was visible in both tested temperatures (38 and 59 °C), but only at 59 °C was a complete disintegration observed after 6 weeks of incubation in mature compost.

PLA is one of the most studied biopolymers for its biodegradation behavior during composting. However, our research shows that some natural biodegradable products made from cellulose also need additional attention, especially when they have chemical additives, as composting could be altered and optimal conditions in composting may not be found. In future observations, these studies should also be extended for molecules—micro and nanoparticles.

In addition, our research shows that composts still bear the capability of degrading biodegradable plastic products if heat is applied externally. Therefore, composts or soils that are polluted with such bioplastics could be treated simply with heat after the composting. This could be an applicable option if such pollutions are discovered in the future.

## Figures and Tables

**Table 1:** Summary of marketed single-use products used in this study.

No	Lining Material	Color	Volume/Size	Weight/g	Wall Thickness/mm
1	PLA	transparent	200	11.6	1.0
2	Sugarcane	transparent	200	6.0	1.2
3	Cellulose	brown	15.5 × 15.5 cm	14	1.3

**Table 2:** Basic physicochemical parameters of compost and biodegradable products.

Samples	MC/%	LOI/%	C/%	N/%	C/N	pH	EC/mS cm^−1^	CEC cmol(+)kg^−1^	NO_3_^−^-N mg kg^−1^ DM	NH_4_^+^-N mg kg^−1^ DM
Compost	62.4 ± 1.6	46.1 ± 0.9	9.21	0.73	14.71	8.69 ± 0.4	3.79 ± 1.02	143.28	1322.03	125.85
PLA	1.9 ± 0.6	98.2 ± 0.6								
Cellulose	2.9 ± 1.0	97.1 ± 1.0								
Sugarcane	4.3 ± 1.4	95.7 ± 1.4								

MC: moisture content; LOI: loss on ignition; CEC: cation exchange capacity; NO_3_-N: nitric nitrogen, NH_4_^+^-N ammoniacal nitrogen; EC: electrical conductivity; DM: dry mass.

**Table 3:** Physicochemical properties of the compost after the composting with biodegradable products process at 38 °C and 59 °C.

Sample	pH, -	Conductivity, mS/cm	MC,%	LOI, %
38 °C				
C	7.64	6.3	39.3 ± 4.5	39.5 ± 2.0
C + PLA	9.28	5.4	39.9 ± 1.2	38.8 ± 1.7
C + CELLULOSE	9.03	4.43	47.8 ± 2.2	37.8 ± 2.2
C + SUGARCANE	8.14	4.46	51.1 ± 0.8	41.2 ± 1.7
59 °C				
C	9.02	6.08	55.3 ± 6.5	33.2 ± 1.6
C + PLA	9.03	5.74	38.8 ± 3.7	35.9 ± 1.0
C + CELLULOSE	8.93	5.67	31.3 ± 2.0	33.3 ± 1.1
C + SUGARCANE	9.06	5.64	46.3 ± 1.2	43.6 ± 6.9

**Table 4:** Weekly weight loss during composting at temperature 38 °C and 59 °C.

Sample Name	Weight Loss, %					
	After 1st Week	After 2nd Week	After 3rd Week	After 4th Week	After 5th Week	After 6th Week
38 °C						
C	26.9 ± 2.6	20.7 ± 1.9	40.7 ± 2.4	52.3 ± 3.0	53.3 ± 3.5	54.7 ± 4.0
C + PLA	14.5 ± 0.9	24.8 ± 1.2	34.8 ± 2.6	41.6 ± 6.0	43.4 ± 4.7	44.3 ± 4.8
C + CELLULOSE	7.1 ± 4.4	17.4 ± 4.8	21.9 ± 5.1	23.3 ± 24.8	24.8 ± 6.0	26.8 ± 6.7
C + SUGARCANE	23.1 ± 4.7	28.8 ± 4.2	32.2 ± 4.4	34.4 ± 5.6	35.4 ± 5.2	36.9 ± 5.2
Evaporation (WATER)			0.99	1.04	1.07	1.08
59 °C						
C	9.4 ± 8.1	29.3 ± 20.4	37.4 ± 23.7	44.6 ± 23.7	54.1 ± 22.6	54.2 ± 22.5
C + PLA	17.1 ± 5.3	33.82 ± 5.3	47.0 ± 6.2	47.0 ± 6.2	47.3 ± 6.3	47.8 ± 6.3
C + CELLULOSE	13.5 ± 15.2	34.7 ± 21.1	42.5 ± 22.0	42.5 ± 22.0	42.9 ± 21.8	43.1 ± 21.7
C + SUGARCANE	2.7 ± 0.3	25.0 ± 14.9	44.3 ± 21	44.3 ± 21.0	44.7 ± 20.8	45.0 ± 20.6
Evaporation (WATER)	2.20	17.00	28.65	35.10	35.23	35.38

**Table 5:** Benchmark of conditions for the composting process to decompose the biopolymers (PLA and cellulose).

Biopolymer	Temperature/MC/Other Parameters	Composting Scale	Time	Results	References
Paper and cellulose mulch (cellulose)	Moisture: 55–65% Temperature >60 °C in 4 weeks C/N ratio—25–30:1	Compost, semi-technical scale	18 weeks	85% biodegradation in 18 weeks	[63]
Organix, BASF ecovio grade M2351, (blend PLA and PBAT)				85–95% biodegradation in 18 weeks	
PLA/PHA				Full biodegradation in 5 weeks	
PLA	Temperature: 58 °C; MC: 60%	Compost, laboratory scale	30 days	Loss of weight 60%	[64]
PLA + clay foil	Temperature: 58 °C; Moisture 55%	Compost, laboratory semi-scale	130 days	biodegradation in PLA 34%	[65]
PLA	Temperature: 58 °C; MC: 48.2% VS: 41.3% pH: 7.9 C/N ratio: 9.9	Compost, laboratory scale	10 month	80% biodegradation in 90 days addition of bioaugmentation	[20]
PLA	Temperature: 65 °C pH: 8.5	Composting, real scale	58 days	84% biodegradation in 58 days	[66]
Cellulose	Temperature: 65 °C pH: 8.5	Composting, real scale	58 days	86% biodegradation in 58 days	[66]

## Data Availability

The data presented in this study are available in the Supplementary Materials.

## Supplementary Materials

The following supporting information can be downloaded at: https://www.mdpi.com/article/10.3390/ma17122948/s1, Table S1. Properties of compost; composition of elements in compost; Figure S1: FTIR results of temperature-treated PLA at 59 °C. (A) FTIR spectra of PLA before (0 weeks) and at 1, 2, 3, 4, 5, and 6 weeks of temperature treatment. (B) Subtracted spectra of samples obtained after 1, 2, 3, 4, 5, and 6 weeks, which were subtracted from the spectra of untreated PLA. The dashed lines indicate zero absorbance change for each subtracted spectrum. (C) Linear slopes of the differences (B) at each wavenumber as the change in the absorbance per week. The horizontal line shows zero absorbance change per week. (D) The coefficient of determination of the linear slopes in (C). The horizontal line shows a threshold of 0.8; Figure S2. FTIR results of temperature-treated PLA at 38 °C. (A) FTIR spectra of PLA before (0 weeks) and at 1, 2, 3, 4, 5, and 6 weeks of temperature treatment. (B) Subtracted spectra of samples obtained after 1, 2, 3, 4, 5, and 6 weeks, which were subtracted from the spectra of untreated PLA. The dashed lines indicate zero absorbance change for each subtracted spectrum. (C) Linear slopes of the differences (B) at each wavenumber as the change in the absorbance per week. The horizontal line shows zero absorbance change per week. (D) The coefficient of determination of the linear slopes in (C). The horizontal line shows a threshold of 0.8; Figure S3. FTIR results of temperature-treated cellulose at 59 °C. (A) FTIR spectra of PLA before (0 weeks) and at 1, 2, 3, 4, 5, and 6 weeks of temperature treatment. (B) Subtracted spectra of samples obtained after 1, 2, 3, 4, 5, and 6 weeks, which were subtracted from the spectra of untreated PLA. The dashed lines indicate zero absorbance change for each subtracted spectrum. (C) Linear slopes of the differences (B) at each wavenumber as the change in the absorbance per week. The horizontal line shows zero absorbance change per week. (D) The coefficient of determination of the linear slopes in (C). The horizontal line shows a threshold of 0.8; Figure S4. FTIR results of temperature-treated cellulose at 38 °C. (A) FTIR spectra of PLA before (0 weeks) and at 1, 2, 3, 4, 5, and 6 weeks of temperature treatment. (B) Subtracted spectra of samples obtained after 1, 2, 3, 4, 5, and 6 weeks, which were subtracted from the spectra of untreated PLA. The dashed lines indicate zero absorbance change for each subtracted spectrum. (C) Linear slopes of the differences (B) at each wavenumber as the change in the absorbance per week. The horizontal line shows zero absorbance change per week. (D) The coefficient of determination of the linear slopes in (C). The horizontal line shows a threshold of 0.8; Figure S5. FTIR results of temperature-treated sugarcane at 59 °C. (A) FTIR spectra of PLA before (0 weeks) and at 1, 2, 3, 4, 5, and 6 weeks of temperature treatment. B) Subtracted spectra of samples obtained after 1, 2, 3, 4, 5, and 6 weeks, which were subtracted from the spectra of untreated PLA. The dashed lines indicate zero absorbance change for each subtracted spectrum. (C) Linear slopes of the differences (B) at each wavenumber as the change in the absorbance per week. The horizontal line shows zero absorbance change per week. (D) The coefficient of determination of the linear slopes in (C). The horizontal line shows a threshold of 0.8; Figure S6. FTIR results of temperature-treated sugarcane at 38 °C. (A) FTIR spectra of PLA before (0 weeks) and at 1, 2, 3, 4, 5, and 6 weeks of temperature treatment. (B) Subtracted spectra of samples obtained after 1, 2, 3, 4, 5, and 6 weeks, which were subtracted from the spectra of untreated PLA. The dashed lines indicate zero absorbance change for each subtracted spectrum. (C) Linear slopes of the differences (B) at each wavenumber as change in the absorbance per week. The horizontal line shows zero absorbance change per week. (D) The coefficient of determination of the linear slopes in (C). The horizontal line shows a threshold of 0.8.
