# Supplementary material for: The Impact of Abiotic and Biotic Conditions for Degradation Behaviors of Common Biodegradable Products in Stabilized Composts
Source: Materials (Basel). 2024 Jun 16;17(12):2948. doi: 10.3390/ma17122948 (PMC11205212; doi:10.3390/ma17122948)
Supplement: Supplementary file 1 [file materials-17-02948-s001.zip › materials-3023431-supplementary.pdf]

Supplementary material

Table S1. Properties of compost; composition of elements in compost

Element	Form	Value
K	total, mg/kg d.m.	17240.91
	leaching, mg/kg d.m.	1218.11
Mg	total, mg/kg d.m.	4082.74
	leaching, mg/kg d.m.	1053.2
Na	total, mg/kg d.m.	456.92
	leaching, mg/kg d.m.	37.66
Ca	total, mg/kg d.m.	27994.05
	leaching, mg/kg d.m.	3188.28
P	total, mg/kg d.m.	4724
	leaching, mg/kg d.m.	749.2
	available, mg/kg d.m.	2852
As	total, mg/kg d.m.	3.64
	available, mg/kg d.m.	0.4
Fe	total, mg/kg d.m.	7999.77
	leaching, mg/kg d.m.	21.24
	available, mg/kg d.m.	27.03
Cu	total, mg/kg d.m.	50.36
	leaching, mg/kg d.m.	3.08
	available, mg/kg d.m.	1.76
Mn	total, mg/kg d.m.	261.18
	leaching, mg/kg d.m.	2.95
	available, mg/kg d.m.	100.97
Al	total, mg/kg d.m.	5053.84
	leaching, mg/kg d.m.	4.02
	available, mg/kg d.m.	65.07
Ni	total, mg/kg d.m.	16.93
	leaching, mg/kg d.m.	3.25
	available, mg/kg d.m.	4.21
Ba	total, mg/kg d.m.	10.14
	leaching, mg/kg d.m.	7.64
	available, mg/kg d.m.	6.9
Cr	total, mg/kg d.m.	13.2
	leaching, mg/kg d.m.	0.08
	available, mg/kg d.m.	0.36

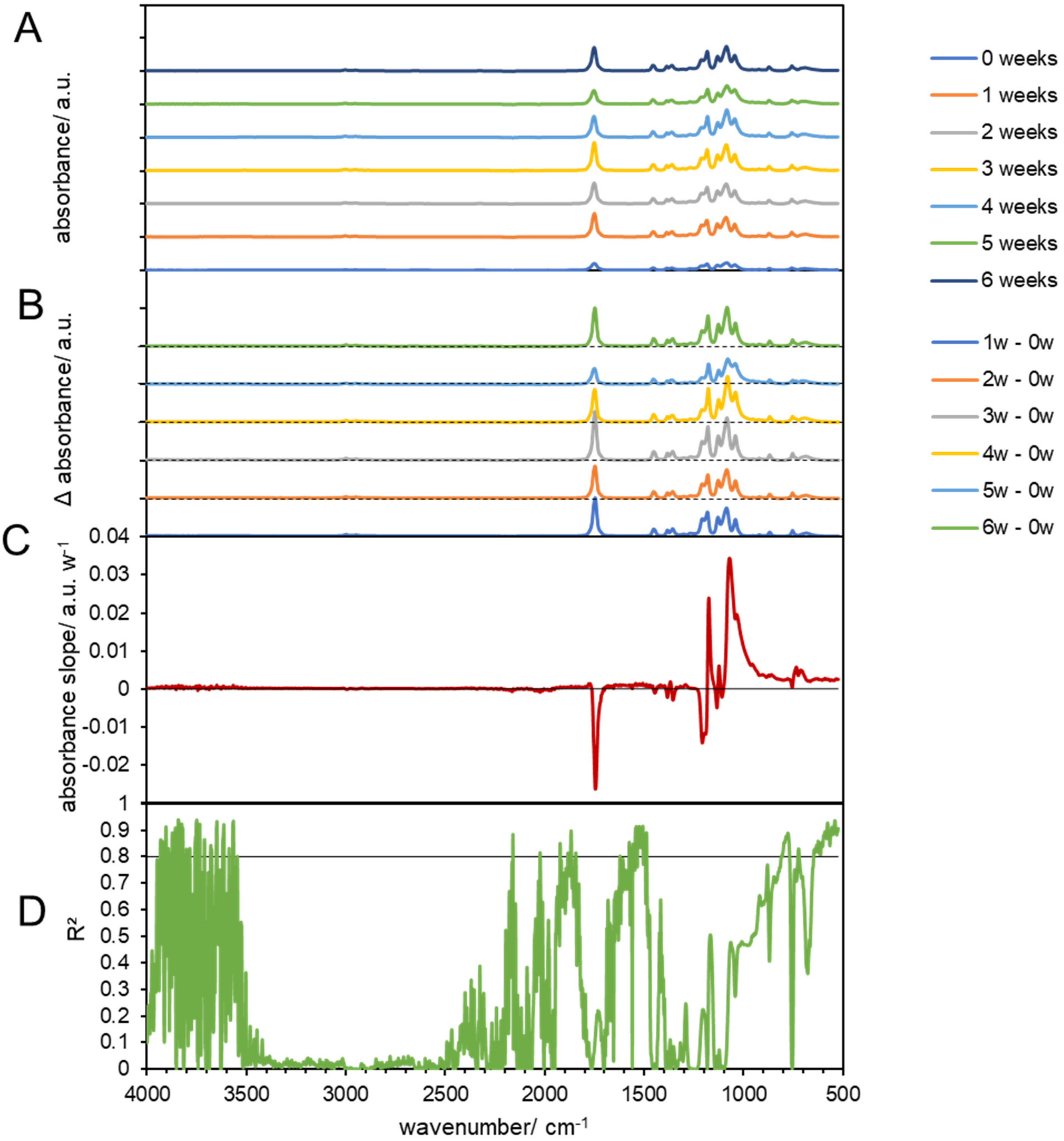

Figure S1: FTIR results of temperature-treated PLA at 59°C. (A) FTIR spectra of PLA before (0 weeks) and at 1, 2, 3, 4, 5, and 6 weeks of temperature treatment. (B) Subtracted spectra of samples obtained after 1, 2, 3, 4, 5, and 6 weeks, which were subtracted from the spectra of untreated PLA. The dashed lines indicate zero absorbance change for each subtracted spectrum. (C) Linear slopes of the differences (B) at each wavenumber as the change in the absorbance per week. The horizontal line shows zero absorbance change per week. (D) The coefficient of determination of the linear slopes in (C). The horizontal line shows a threshold of 0.8.

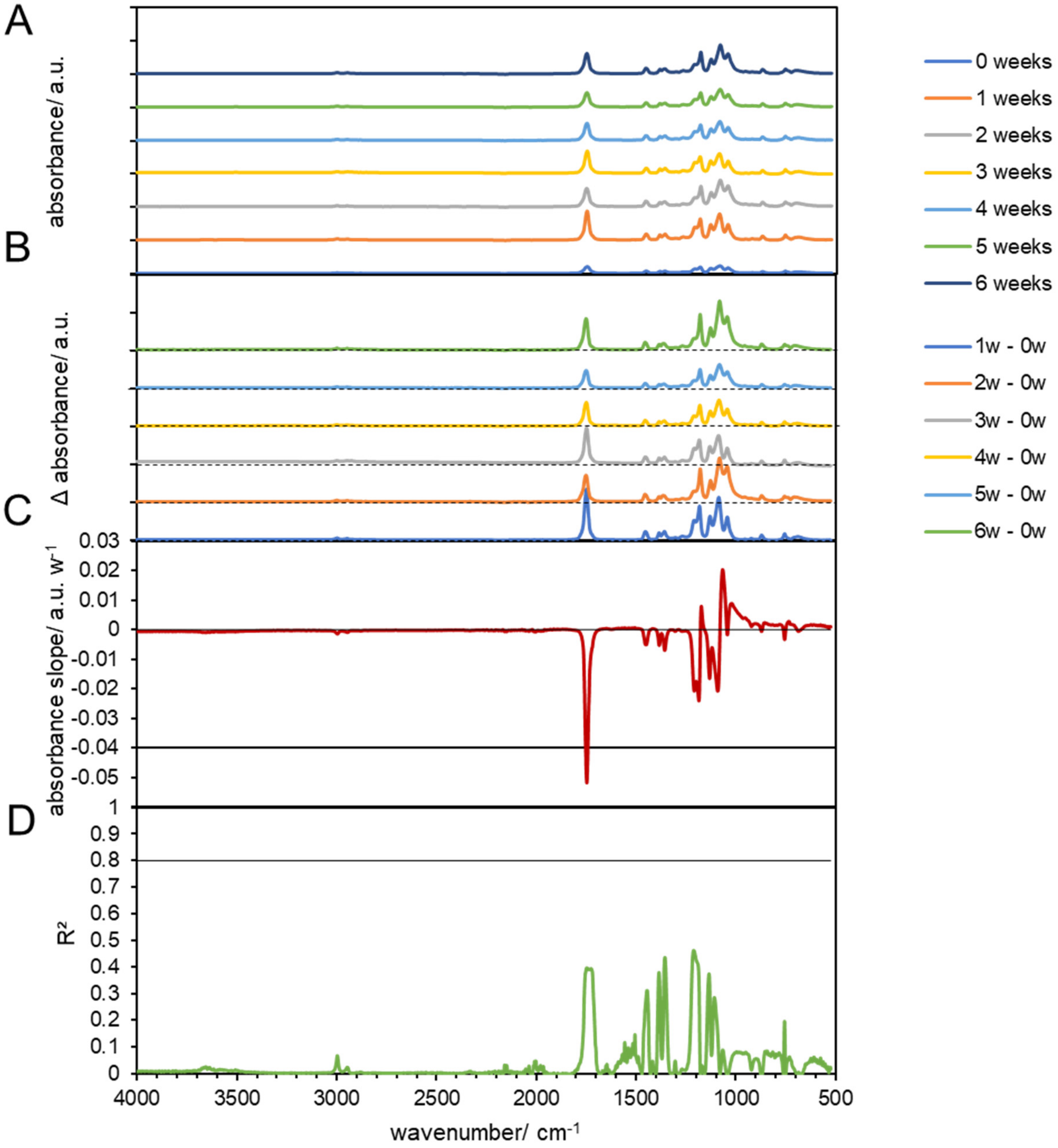

Figure S2: FTIR results of temperature-treated PLA at 38°C. (A) FTIR spectra of PLA before (0 weeks) and at 1, 2, 3, 4, 5, and 6 weeks of temperature treatment. (B) Subtracted spectra of samples obtained after 1, 2, 3, 4, 5, and 6 weeks, which were subtracted from the spectra of untreated PLA. The dashed lines indicate zero absorbance change for each subtracted spectrum. (C) Linear slopes of the differences (B) at each wavenumber as the change in the absorbance per week. The horizontal line shows zero absorbance change per week. (D) The coefficient of determination of the linear slopes in (C). The horizontal line shows a threshold of 0.8.

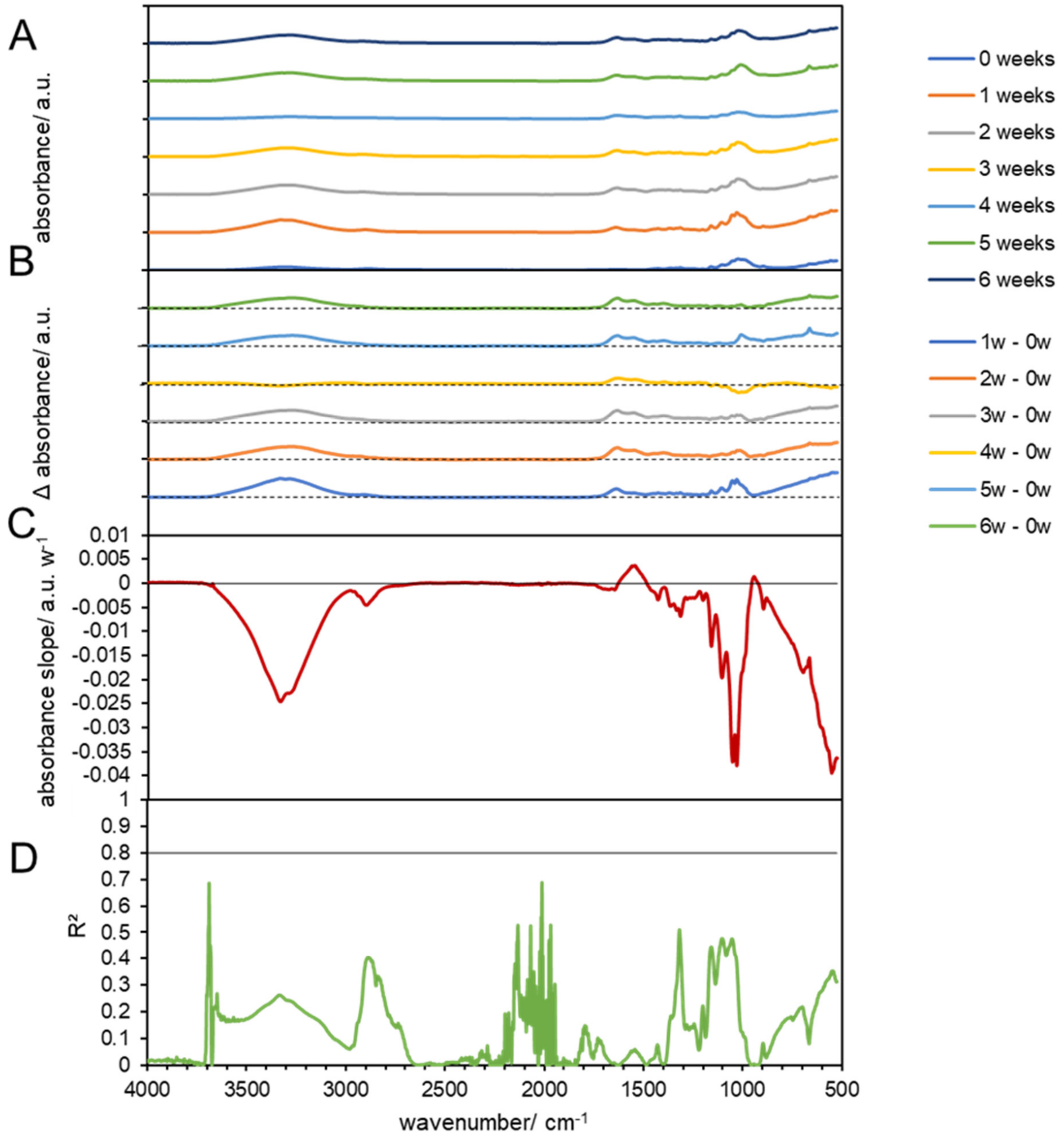

Figure S3: FTIR results of temperature-treated cellulose at 59°C. (A) FTIR spectra of PLA before (0 weeks) and at 1, 2, 3, 4, 5, and 6 weeks of temperature treatment. (B) Subtracted spectra of samples obtained after 1, 2, 3, 4, 5, and 6 weeks, which were subtracted from the spectra of untreated PLA. The dashed lines indicate zero absorbance change for each subtracted spectrum. (C) Linear slopes of the differences (B) at each wavenumber as the change in the absorbance per week. The horizontal line shows zero absorbance change per week. (D) The coefficient of determination of the linear slopes in (C). The horizontal line shows a threshold of 0.8.

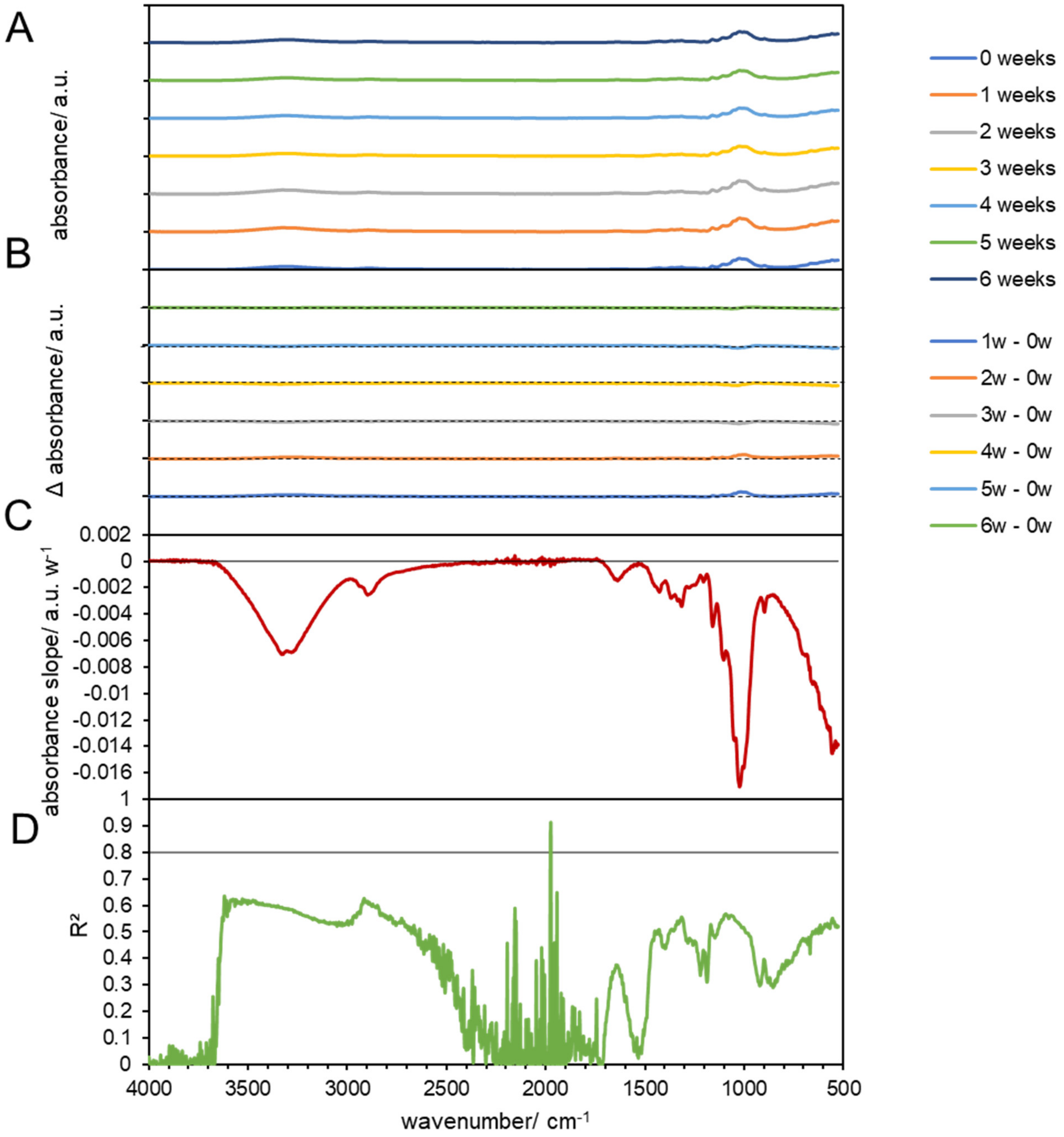

Figure S4: FTIR results of temperature-treated cellulose at 38°C. (A) FTIR spectra of PLA before (0 weeks) and at 1, 2, 3, 4, 5, and 6 weeks of temperature treatment. (B) Subtracted spectra of samples obtained after 1, 2, 3, 4, 5, and 6 weeks, which were subtracted from the spectra of untreated PLA. The dashed lines indicate zero absorbance change for each subtracted spectrum. (C) Linear slopes of the differences (B) at each wavenumber as the change in the absorbance per week. The horizontal line shows zero absorbance change per week. (D) The coefficient of determination of the linear slopes in (C). The horizontal line shows a threshold of 0.8.

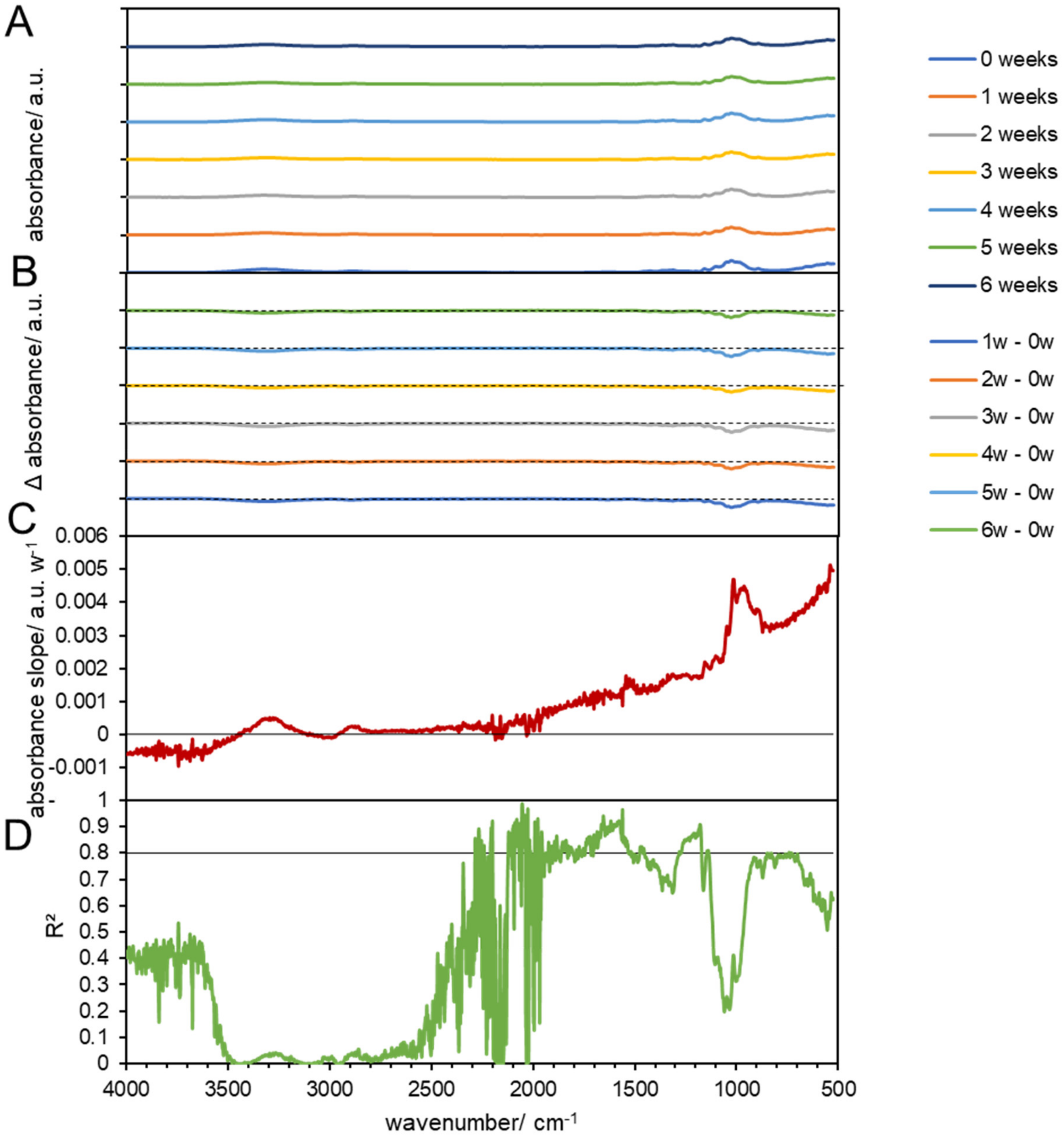

Figure S5: FTIR results of temperature-treated sugarcane at 59°C. (A) FTIR spectra of PLA before (0 weeks) and at 1, 2, 3, 4, 5, and 6 weeks of temperature treatment. (B) Subtracted spectra of samples obtained after 1, 2, 3, 4, 5, and 6 weeks, which were subtracted from the spectra of untreated PLA. The dashed lines indicate zero absorbance change for each subtracted spectrum. (C) Linear slopes of the differences (B) at each wavenumber as the change in the absorbance per week. The horizontal line shows zero absorbance change per week. (D) The coefficient of determination of the linear slopes in (C). The horizontal line shows a threshold of 0.8.

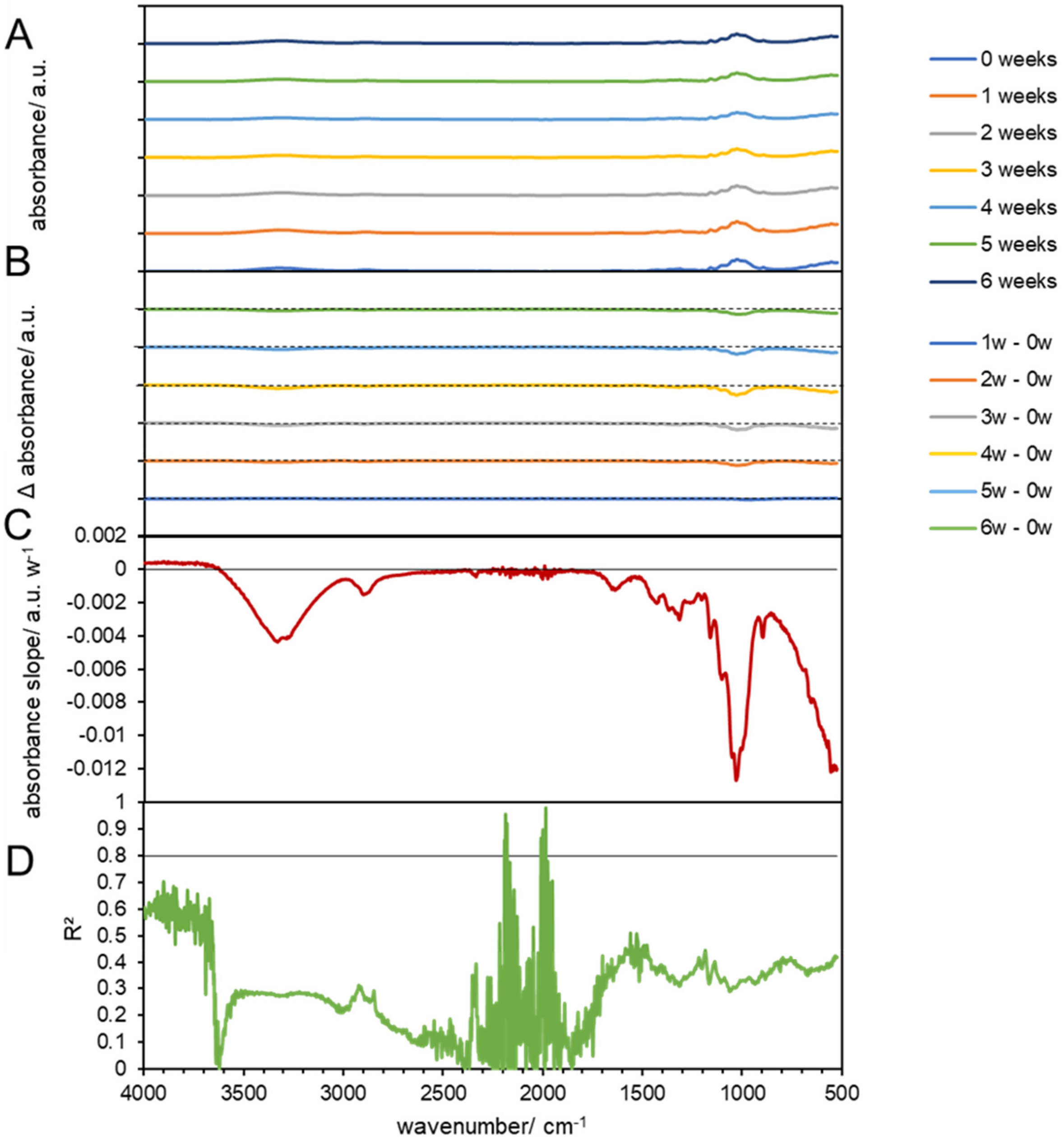

Figure S6. FTIR results of temperature-treated sugarcane at 38°C. (A) FTIR spectra of PLA before (0 weeks) and at 1, 2, 3, 4, 5, and 6 weeks of temperature treatment. (B) Subtracted spectra of samples obtained after 1, 2, 3, 4, 5, and 6 weeks, which were subtracted from the spectra of untreated PLA. The dashed lines indicate zero absorbance change for each subtracted spectrum. (C) Linear slopes of the differences (B) at each wavenumber as the change in the absorbance per week. The horizontal line shows zero absorbance change per week. (D) The coefficient of determination of the linear slopes in (C). The horizontal line shows a threshold of 0.8.
